# Function of anterior talofibular and calcaneofibular ligaments during in-vivo motion of the ankle joint complex

**DOI:** 10.1186/1749-799X-4-7

**Published:** 2009-03-16

**Authors:** Richard J de Asla, Michal Kozánek, Lu Wan, Harry E Rubash, Guoan Li

**Affiliations:** 1 The Bioengineering Laboratory, Department of Orthopaedic Surgery, Massachusetts General Hospital and Harvard Medical School, 55 Fruit Street – GRJ 1215, Boston, MA 02114, USA

## Abstract

**Background:**

Despite the numerous in-vitro studies on the mechanical properties and simulated injury mechanisms of the anterior talofibular ligament (ATFL) and calcaneofibular ligament (CFL), the in-vivo biomechanical behavior of these two ligaments has not yet been described.

**Methods:**

Apparent length of the ATFL and CFL was measured in four ankles in healthy male subjects between 32 and 45 years of age (two left and two right) during a dorsiflexion-plantarflexion and supination-pronation arc of motion using a combined dual-orthogonal fluoroscopic and magnetic resonance imaging technique.

**Results:**

The ATFL elongated from the neutral position at 16.3 +/- 3.0 mm to 20.8 +/- 2.7 mm at maximal plantarflexion and shortened significantly from the neutral position to 13.9 +/- 2.9 mm at maximal dorsiflexion (*p *= 0.01). The CFL shortened from the neutral position at 28.0 +/- 2.9 mm to 26.6 +/- 2.2 mm at maximal plantarflexion (*p *= 0.08) and elongated significantly from the neutral position to 29.9 +/- 3.0 mm at maximal dorsiflexion (*p *= 0.003). The ATFL elongated significantly from 14.8 +/- 2.5 mm at maximal pronation to 17.4 +/- 3.0 mm at maximal supination (*p *= 0.08). At the same time, the CFL shortened from 31.0 +/- 3.8 mm at maximal pronation to 26.9 +/- 3.6 mm at maximal supination (*p *= 0.02).

**Conclusion:**

The results showed that the ATFL elongates more during plantarflexion and supination whereas the CFL increases in length with dorsiflexion and pronation. Concurrently, these data also demonstrated the reciprocal function between the two ligaments. While one shortens, the other one elongates. The different elongation of the ATFL and CFL during the same motion arc suggests that under excessive loading conditions the ATFL might be more vulnerable in plantarflexion and supination while the CFL might be more susceptible to injury in dorsiflexion and pronation. Furthermore, in the case of surgical reconstruction the grafts used to reconstruct the two ligaments may need to be tensioned at different positions of the ankle in order to reproduce their natural in vivo function.

## Introduction

Inversion injuries to the ankle are among the most common problems in musculoskeletal care, representing 10% of all visits to the emergency room. [1,2] The incidence of inversion ankle injuries is reported as one in 10,000 people per day.[1,2] Up to 20% of patients sustaining an inversion injury to the ankle will experience persistent symptoms such as functional instability, recurrent sprains or chronic pain.[3] The anterior talofibular ligament (ATFL) and the calcaneofibular ligament (CFL) are the most commonly involved lateral ankle ligaments.[4]

Numerous studies have investigated the mechanical properties and simulated injury mechanisms of the ATFL and CFL in-vitro and much has been learned regarding ligament morphology [5-8] and tensile strength[9] or strain changes in-vitro.[7,10] However, the in-vivo behavior of these two ligaments is still not well studied. To the best of our knowledge, no data has been reported on the elongation of the ATFL and CFL in-vivo. The objective of the current study was to measure the change in apparent length of the ATFL and CFL, in-vivo, in uninjured healthy ankles during a dorsiflexion-plantarflexion and supination-pronation arc of motion using a combined dual-orthogonal fluoroscopic and magnetic resonance (MR) imaging technique.[11]

## Materials and methods

### Subject recruitment

Four ankles of healthy male subjects between 32 and 45 years of age (two left and two right, average height of 175 cm and average body mass index (BMI) of 21.8 kg/m^2^) were included in this study. None of the participating subjects had subjective complaints of ankle pain, previous history of ankle injury requiring treatment, systemic disease, or contraindication for MR imaging. Prior to MR scanning, both ankles, including the scanned and the contralateral one, were evaluated by an orthopaedic surgeon in order to rule out pathology.

The purpose of the study was explained in detail to all participants. An Institutional Review Board approved consent form was obtained from each subject prior to participation in the study.

### Three dimensional model of the ankle joint complex (AJC)

**Figure 1:**
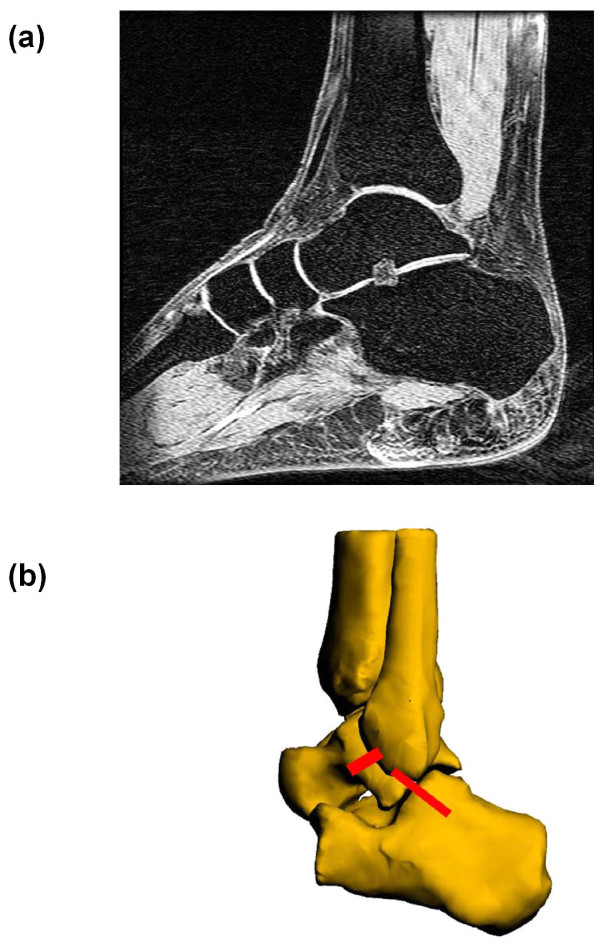
**(a) MR image of the ankle joint complex (AJC) used to create a three-dimensional virtual model (b) 3D model of the AJC with anterior talofibular ligament (ATFL) and calcaneofibular ligament (CFL)**.

Each ankle was MR imaged on a 1.5 T scanner (GE, Milwaukee, WI) using a surface coil and three-dimensional spoiled gradient-recalled echo (3D SPGR) sequence with the subject lying in a relaxed, supine position. During the MR scanning the ankle was held in a brace with the foot at a right angle with respect to the shaft of the tibia. This position was considered a neutral reference position. A field of view (16 × 16 × 10 cm^3^) that spanned the medial and lateral boundaries of the ankle joint complex (AJC) (which we define as the distal tibia, fibula, talus, calcaneus, the tibiotalar joint, and subtalar joint) was created from the MR scan with 16 cm in both anterior-posterior and proximal-distal directions and 10 cm in the medial-lateral direction. Parallel, sagittal, coronal and axial images with a resolution of 512 × 512 pixels were taken, separated at 1 mm intervals (a sample sagittal MR slice is shown on Figure 1a). The sagittal MR images were imported into solid modeling software (Rhinoceros^®^, Robert McNeel and Associates, Seattle, WA) and digitized to outline the contours of tibia, fibula, talus, and calcaneus. Afterwards, these outlines were used to reconstruct 3D geometric models of the AJC (Figure 1b) [11-13]. The ligament attachment sites were determined from the MR images with the assistance of anatomical studies.[5,14] Once the MR images of each studied AJC were obtained, they were imported into virtual environment of the solid modeling software where the insertions were digitally outlined on each MR slice. Thereafter, an orthopedic surgeon specializing in foot and ankle surgery as well as a musculoskeletal radiologist verified the outlined insertions on each studied AJC. Furthermore, in preparation for this study we also dissected four cadaveric ankle specimens and meticulously identified the insertion site anatomy of ATFL and CFL.

### Dual-orthogonal fluoroscopic imaging

Two fluoroscopes (OEC^® ^9800 ESP, GE, Salt Lake City, UT) were positioned in planes orthogonal to each other in order to capture simultaneous images of the AJC from two orthogonal projections (anterolateral and anteromedial). The fluoroscopic images have a resolution of 1000 × 1000 pixels. During the experiment, the subject stood upright with one leg on a platform and positioned the other (studied) ankle within the common imaging zone of the two fluoroscopes. This was done with the assistance of laser positioning devices embedded in the fluoroscopes. Each ankle was then tested in four non-weightbearing positions: maximal plantarflexion (rotation around the medial-lateral axis with the foot turning downward), maximal dorsiflexion (rotation around the medial-lateral axis with the foot turning upward), maximal supination (foot position that combines inversion, plantar flexion and internal rotation) and maximal pronation (combination of eversion, dorsal flexion and external rotation). During these non-weightbearing tests, the subject lifted the testing foot in view of both fluoroscopes and actively performed the target motion.

### Virtual reproduction of in-vivo AJC motion

**Figure 2:**
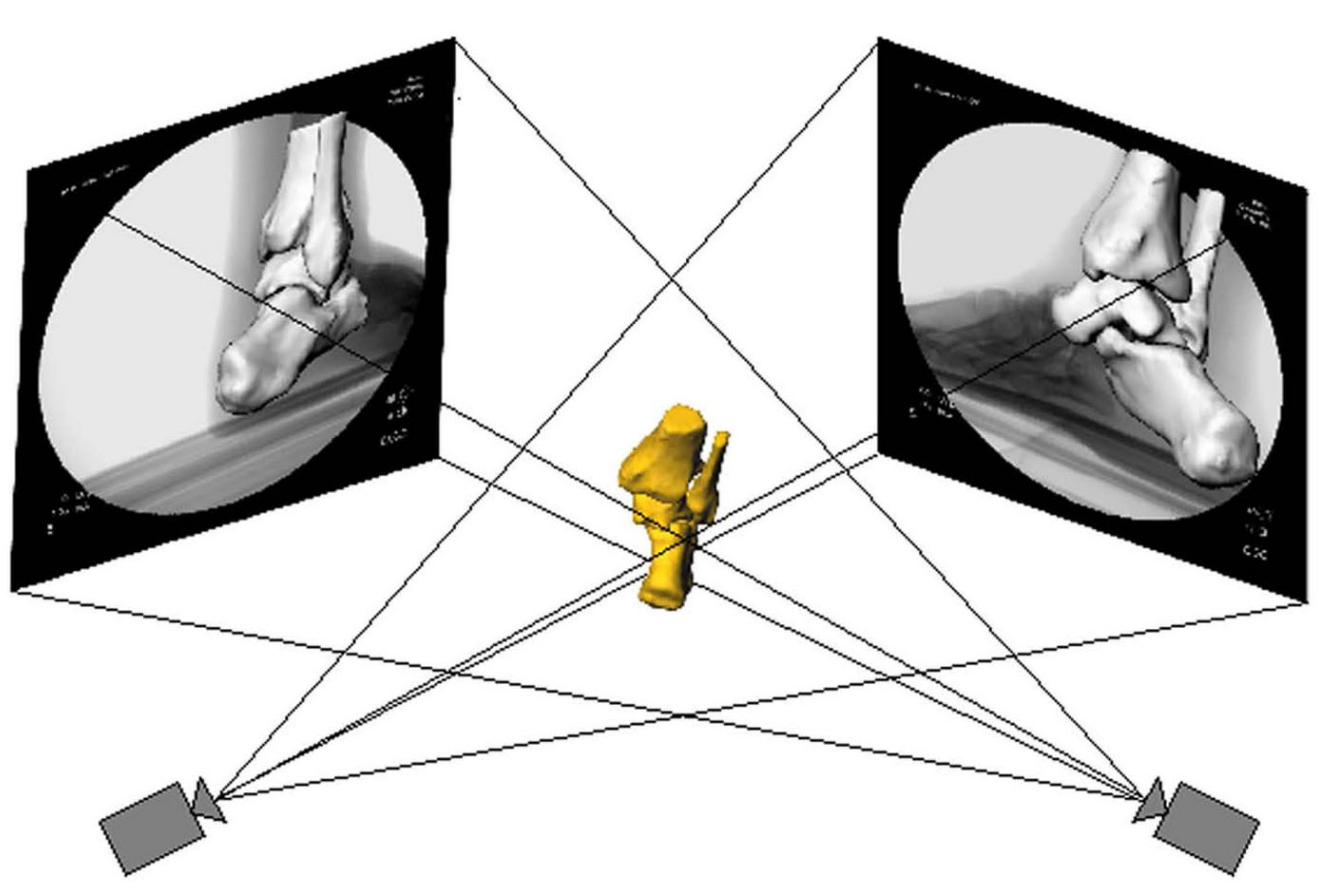
**Reproduction of the in-vivo AJC position using the virtual dual-orthogonal fluoroscopic system setup**.

The three-dimensional (3D) model of the AJC and the fluoroscopic images were imported into the solid modeling software to create a virtual dual-orthogonal fluoroscopic system. Two virtual cameras were created to represent the X-ray sources of the two fluoroscopes. The orthogonal fluoroscopic images were then placed to reproduce the position of the two intensifiers of the fluoroscopes. Afterwards, the 3D AJC model was imported into the virtual space and the position of the tibia, fibula, talus, and calcaneus were each adjusted separately in six degrees of freedom (6DOF) under visual control until the projected outlines of the AJC model matched the actual AJC on the fluoroscopic images (Figure 2). [11] The process was repeated for each AJC position. The method has been rigorously validated and has an accuracy of 0.1 mm in translation and 0.18 degrees in rotation.[15]

### Measurement of apparent length of the ligaments

The apparent lengths of the ligaments were defined as the shortest distances between the centroids of the digitized attachment areas on talus, calcaneus and fibula. Once the position of the AJC was reproduced in the modeling software, the length of the ATFL and CFL was measured for each testing position of AJC motion. There was no bony interference with the path of the ATFL and CFL. The position of the AJC during MR scanning was defined as the neutral reference position. The length of the ATFL and CFL was compared from dorsiflexion to plantar flexion and from supination to pronation. The neutral position was used as a reference in each comparison.

### Data analysis

A Friedman's test was used to statistically compare the differences between the lengths of the ligaments at each tested position of the AJC. The level of significance was set at *p *< 0.05.

## Results

### Maximal dorsiflexion and maximal plantarflexion

**Figure 3:**
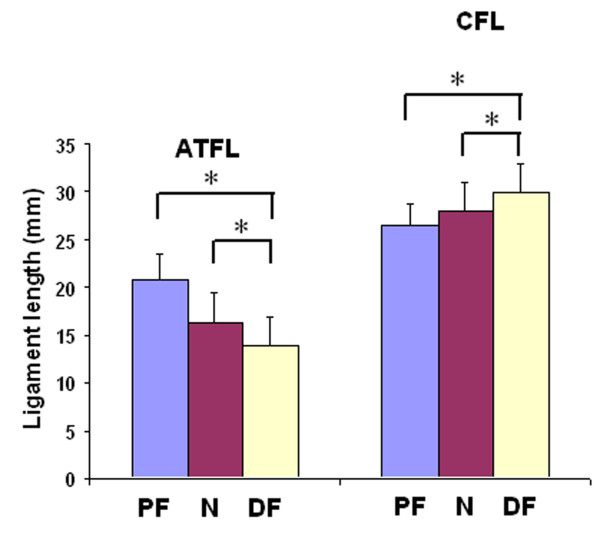
**Lengths of the ATFL and CFL as measured at maximal plantarflexion (PF), maximal dorsiflexion (DF) and at neutral position (N)**. *p < 0.05.

The ATFL elongated from the neutral position at 16.3 ± 3.0 mm to 20.8 ± 2.7 mm at maximal plantarflexion and shortened significantly from the neutral position to 13.9 ± 2.9 mm at maximal dorsiflexion (*p *= 0.01), as shown in Figure 3. The ATFL also elongated significantly from maximal dorsiflexion to maximal plantarflexion (*p *= 0.049). The average change in length of the ATFL from maximal dorsiflexion to maximal plantarflexion was therefore 7 mm.

The CFL shortened from the neutral position at 28.0 ± 2.9 mm to 26.6 ± 2.2 mm at maximal plantarflexion (*p *= 0.08) and elongated significantly from the neutral position to 29.9 ± 3.0 mm at maximal dorsiflexion (*p *= 0.003), as shown in Figure 3. The average change in length of the CFL during the maximal dorsiflexion to maximal plantarflexion arc of motion was 3 mm.

### Maximal supination and maximal pronation

**Figure 4:**
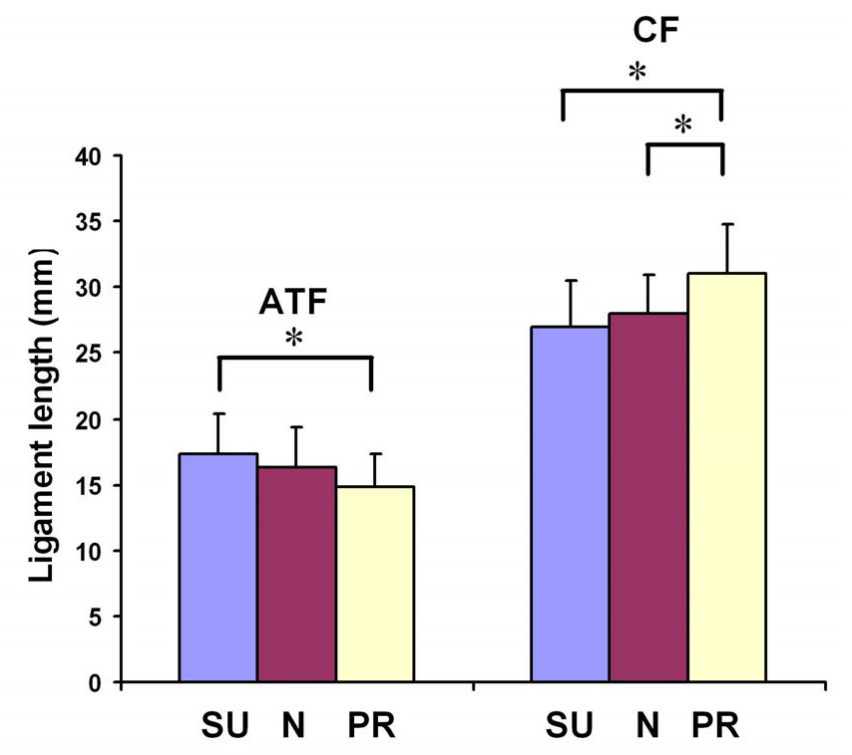
**Lengths of the ATFL and CFL as measured at maximal supination (SU), maximal pronation (PR) and at neutral position (N)**. *p < 0.05.

The ATFL elongated from the neutral position to 17.4 ± 3.0 mm at maximal supination (*p *= 0.09) and shortened from neutral position to 14.8 ± 2.5 mm at maximal pronation (*p *= 0.08; Figure 4). It also elongated significantly from maximal pronation to maximal supination (*p *= 0.006). The average change in length of the ATFL from maximal supination to maximal pronation was 2.6 mm.

The CFL shortened from the neutral position to 26.9 ± 3.1 mm at maximal supination (*p *= 0.07) and elongated significantly from the neutral position to 31.0 ± 3.8 mm at maximal pronation (*p *= 0.04), as shown in Figure 4. The CFL also significantly elongated from maximal supination to maximal pronation (*p *= 0.02). The average change in length of the CFL from maximal supination to maximal pronation was 4.1 mm.

## Discussion

The present study investigated the changes in length of the ATFL and CFL in-vivo during the positions of plantarflexion, dorsiflexion, supination and pronation using a combined MR and dual-orthogonal fluoroscopy. The results showed that the ATFL is significantly more elongated at plantarflexion than at dorsiflexion. Further, the ATFL is also significantly more elongated at supination than at pronation. Conversely, the CFL is more elongated with dorsiflexion than with plantarflexion and was also found to be more elongated at pronation than at supination. Therefore, these data also demonstrated the reciprocal function between the two ligaments. While one shortens, the other one elongates and vice versa.

Several cadaveric studies investigated the length of lateral ankle ligaments. Milner et al.[6] measured length of osteoligamentous preparations of collateral ligaments in 40 ankle specimens and reported the length of the ATFL and CFL to be 13.0 ± 3.9 mm and 19.5 ± 3.9 mm respectively. Siegler et al.[8] reported the average lengths of ATFL and CFL measured in osteoligamentous preparations of 20 cadaveric ankles. In their study, the average ATFL length was 17.8 ± 3.1 mm and the average CFL length was 27.7 ± 3.3 mm. Burks and Morgan also measured the length of lateral ankle ligaments in their sample of 39 cadaveric ankles.[5] They reported the average length of the ATFL and CFL as 24.8 mm and 35.8 mm respectively. The in-vivo length of the ATFL and CFL measured in this study in a neutral position compares favorably with the results of Milner et al.[6] and Siegler et al.[8] who measured the distance between the ligament attachment sites. In this study the average length of the ATFL was 15.8 ± 2.9 mm and the average length of the CFL was 27.7 ± 2.7 mm in the neutral position. The study of Burks and Morgan measured the length of the longest fibers, while our study used centroids of insertion areas, which might explain why their values were larger. Burks and Morgan also showed that the ATFL and the CFL have adjacent attachment sites on the anterior edge of the fibula 8–10 mm from the distal tip and that with the foot in neutral position, the CFL forms an angle of about 130 degrees with the fibula whereas the ATFL was slightly less than 90 degrees anteriorly.[5] It was therefore theorized that in dorsiflexion, the CFL assumes a course parallel to the axis of the fibula thereby functioning as a collateral ligament. In plantarflexion, the orientation of ATFL fibers assumes this position and may be expected to function as the main collateral ligament.[5,16] Since the majority of sprains occur during plantarflexion, [4,17] the ATFL would therefore be the first ligament to suffer disruption in this type of injury, as shown in several reports.[4,18,19] This notion was supported by numerous in-vitro studies which investigated the changes in ATFL and CFL strain in response to loading in various positions. Colville et al.[10] measured strain in lateral ankle ligaments in 10 cadaveric ankles while applying inversion/eversion and internal/external rotational forces during the flexion-extension arc of motion. They demonstrated increased strain in the ATFL with increasing plantarflexion, inversion and internal rotation. Strain in the CFL was found to increase in dorsiflexion and inversion. Ozeki et al.[7] investigated strain changes in central fibers of the lateral ankle ligaments in 12 cadaveric specimens during plantarflexion-dorsiflexion. Their results showed that ATFL was taut in plantarflexion and the CFL in dorsiflexion. The length change of each ligament during the arc of motion was less than 1.6 mm. Renström et al.[20], Rasmussen[21,22] and Bahr et al.[16] also found that the ATFL acts as a primary restraint in inversion and plantarflexion, whereas the CFL tension was increased mainly in inversion and dorsiflexion. The lowest maximum load-to-failure of ATFL among the lateral ankle ligaments also explains why ATFL is often the first ligament to rupture during an inversion injury to the ankle.[23] These cadaveric studies have provided fundamental knowledge for the understanding of function of these ligaments and injury mechanisms. However, the in-vivo behavior of the lateral ankle ligaments during the range of motion has not been well studied.

Data in this study demonstrate that the ATFL lengthens more in plantarflexion and supination (combination of plantarflexion, inversion and internal rotation) whereas the CFL elongates more in dorsiflexion and pronation (combination of dorsiflexion, eversion and external rotation). These data compare favorably with the results of previous cadaveric studies and suggest that under excessive loading conditions the ATFL might be more vulnerable in plantarflexion and supination while the CFL might be more susceptible to injury in dorsiflexion and pronation.

Certain limitations of this study should be noted. The ankles were studied during non-weightbearing motion and the subjects were asked to actively position their ankles into the extreme positions of plantarflexion, dorsiflexion, supination, and pronation. These ranges of motion are most likely smaller than what would be measured if the foot was passively moved and held in position. Additionally, the volunteers may not have applied the supination pronation motion in the same manner. However, we tried to minimize the potential inconsistencies by instructing the subjects about the target positions and visually verifying them during the scanning. It should be noted that this study did not investigate motion of the ligaments, but estimated the changes in length based on the distances between the centroids of the digitized attachment sites. The methodology has not been validated to measure in-vivo motion of the ligaments. Further, this study only included male subjects since it has been shown that male and female ankles and feet differ in several anthropometric characteristics.[24,25] Therefore, in the future, ligament function should be investigated in female subjects. Finally, only four positions were studied (full plantarflexion, dorsiflexion, supination and pronation).

In conclusion, the results of this study contribute to the small pool of data on in-vivo behavior of the lateral ankle ligaments. We noted that the ATFL seems to elongate more during plantarflexion and supination whereas the CFL increases in length with dorsiflexion and pronation. Concurrently, these data also demonstrated the reciprocal function of the two ligaments. While one shortens, the other one elongates. The different elongation of the ATFL and CFL during the same motion arc suggests that under excessive loading conditions the ATFL might be more vulnerable in plantarflexion and supination while the CFL might be more susceptible to injury in dorsiflexion and pronation. Furthermore, in the case of surgical reconstruction the grafts used to reconstruct the two ligaments may need to be tensioned at different positions of the ankle in order to reproduce their natural in vivo function. In the future it will be possible to apply this technique to study the ligament function after various types of injury and to evaluate the effectiveness of operative or conservative treatment in restoring normal ligament behavior in vivo. Furthermore, dynamic motion of the ankle should be studied.

## Competing interests

The authors declare that they have no competing interests.

## Authors' contributions

All authors were directly involved in the experiments, data analysis, interpretation of results and preparation of the manuscript. All authors have reviewed the text of the manuscript and agree with publication in the present form. RJD carried out scanning, recruited subjects, performed data analysis, prepared manuscript, revised manuscript. MK carried out scanning, subject recruitment, image processing, preparation of the manuscript and editing. LW assisted with scanning, subject recruitment, image processing and data analysis. HR supervised data analysis and interpretation, advised co-authors in preparation and revision of the manuscript. GL designed experiment, supervised data analysis and manuscript preparation and revision.
